# A novel dietary inflammatory index reflecting for inflammatory ageing: Technical note

**DOI:** 10.1016/j.amsu.2019.09.012

**Published:** 2019-09-27

**Authors:** Masao Kanauchi, Mitsuru Shibata, Masaki Iwamura

**Affiliations:** a Department of Health and Nutrition, Faculty of Health Science, Kio University, Koryo-cho, Kitakatsuragi District, Nara, Japan; b Department of Physical Therapy, Faculty of Health Science, Aino University, Higashioda, Ibaraki City, Japan

**Keywords:** Chronic inflammation, Dietary inflammatory index, Mediterranean diet

## Abstract

**Objective:**

Chronic inflammation plays an important role in the development of several chronic diseases. Existing dietary inflammatory indexes require complicated calculations, which are difficult to use in clinical practice. We developed a new and simple index, based solely on the frequency of consumption of only 16 foods, to capture the inflammatory potential of diet.

**Methods:**

The new index, an empirical dietary inflammatory index (eDII), is based on 8 pro-inflammatory and 8 anti-inflammatory components. First, in a validation study, 168 community-dwelling persons were invited to participate and an inflammatory aging disease (IAD) score of each participant was calculated by total number of IADs. Second, in the nutritional epidemiologic study, we calculated the eDII for 1464 participants and compared the eDII with healthy diet quality scores.

**Results:**

In a validation study, when subjects were classified by eDII tertile, a higher eDII was significantly associated with a higher IAD score. In the nutritional epidemiologic study, a higher eDII was inversely associated with the Mediterranean diet score, the World Health Organization's healthy diet indicator, and the American Heart Association's recommended healthy diet score.

**Conclusions:**

The eDII is an easy and valid instrument to assess the inflammatory potential of dietary factors. This index is easy to use and does not require detailed estimations of nutrient intake.

## Highlights

• We developed a new and simple index to capture the inflammatory potential of diet.
• The new index is based on 8 pro-inflammatory and 8 anti-inflammatory components.
• This index is related to an inflammatory aging disease score.
• This index is easy to use and does not require detailed estimations of nutrient intake.

## 1 Introduction

Chronic inflammation plays an important role in the development of several chronic diseases [1]. Because various nutrients and foods have been shown to modulate inflammation, habitual dietary patterns may play a role in the regulation of chronic inflammation [2]. One of the widely used indexes to assess the inflammatory potential of dietary factors is the dietary inflammatory index (DII), which is based on 45 dietary factors including macronutrients, vitamins, minerals, flavonoids, spices, and herbs [3]. Another DII based on 9 pro-inflammatory and 9 anti-inflammatory foods was also developed [4]. However, both indexes were developed using daily amounts of food or nutrient consumption and require complicated calculations, which are difficult to use in clinical practice. We developed a new and simple index, based solely on the frequency of consumption of only 16 foods, to capture the inflammatory potential of diet.

## 2 Materials & methods

The new index, an empirical dietary inflammatory index (eDII), is based on 8 pro-inflammatory components (red meats, processed meats, organ meats, other fish, eggs, sugar-sweetened beverages, tomatoes, and refined grains) and 8 anti-inflammatory components (leafy green vegetables, dark yellow vegetables, fruit juice, oily fish, coffee, tea, wine, and beer or other alcohol beverages). Food components of our eDII are based on the DII proposed by Tabung et al. [4] with minor modifications. However, our eDII is different from the conventional DII, which uses calculations based on daily amounts of foods or nutrient consumption. In contrast, our eDII is based on the frequency of consumption of various foods, that is, how many times per week (or how many bowls or cups per day) a subject consumed. The framework for the scoring system of the eDII was designed using the Mediterranean diet pyramid [5] and its related literature [6]. Each pro-inflammatory component was scored 0, 1, or 2 points, and anti-inflammatory components was scored 0, -1, or -2 points (Table 1). Exceptionally, we set an upper limit for alcohol consumption based on data from the dose-response meta-analysis [7] because excessive alcohol is inflammatory. Total scores ranged from −16 to +16, with a higher score indicating a higher inflammatory potential.

**Table 1:** Scoring system of the empirical dietary inflammatory index.

Pro-inflammatory foods	+2 points	+1 point	0 points
Red meat, processed meat, organ meat	≥7 times/w	2-6 times/w	<2 times/w
Other fish, eggs, SSB, tomatoes	≥7 times/w	5-6 times/w	<5 times/w
Refined grains			
White rice	**-**	≥3 bowls/d	<3 bowls/d
Bread/noodles	**-**	≥7 times/w	<7 times/w
**Anti-inflammatory foods**	**−2 points**	**−1 point**	**0 points**
Leafy green vegetables	≥14 times/w	7-13 times/w	<7 times/w
Dark yellow vegetables	≥7 times/w	5-6 times/w	<5 times/w
Fruit juice, oily fish	≥5 times/w	2-4 times/w	<2 times/w
Coffee, tea	≥2 cups/d	1 cup/d	<1 cup/d
Wine	7-20 glasses/w	2-6 glasses/w	<2 glasses/w, ≥21 glasses/w
Beer or other alcohol beverages	7-13 bottles/w	5-6 bottles/w	<5 bottles/w, ≥14 bottles/w

SSB, sugar sweetened beverages.

First, in a validation study, 168 community-dwelling persons (104 men and 64 women) were invited to participate in the Kashiba Silver Agers Healthy Longevity Study (KASAHeL Study, Kashiba, Japan). The inflammatory aging disease (IAD) score of each participant was calculated by total number of IADs: cardiovascular disease, stroke, cancer, chronic obstructive pulmonary disease, chronic kidney disease, diabetes, obesity, dementia, depression, and sarcopenia. Chi-square test was used to compare distribution of IAD score by eDII tertile.

Second, in nutritional epidemiologic study, we calculated the eDII for 1464 participants (788 men and 676 women) from 8 workplaces, 1 university, and 3 communities from our pervious studies [[8], [9], [10]]. We compared the eDII with their healthy diet quality scores: the Mediterranean diet score adapted to Japanese (jMDS) [8], the World Health Organization's healthy diet indicator (WHO-HDI) [9], and the American Heart Association's recommended healthy diet score (AHA-HDS) [10].

## 3 Results

In a validation study, the eDII scores in our subjects ranged from −7 to +8, and scores were distributed symmetrically (data not shown). The proportion of higher IAD score significantly increased across eDII tertile (p = 0.018) (Table 2).

**Table 2:** The IAD distribution across tertile of the eDII.

	T1	T2	T3	P *
Score range	−7 to −2	−1 to +1	+2 to +8	0.018
IAD score 0	27 (52.9%)	23 (35.9)	11 (20.8)	
IAD score 1	13 (25.5)	21 (32.8)	20 (37.7)	
IAD score ≥2	11 (21.6)	20 (31.3)	22 (41.5)	

Data are n (%).

* by chi-square.

IAD, inflammatory aging disease; eDII, empirical Dietary Inflammatory Index.

In the nutritional epidemiologic study, we compared the eDII to three healthy diet scores. According to the low, moderate, and high range of the eDII, a higher eDII was significantly associated with a lower jMDS, WHO-HDI, and AHA-HDS score (Table 3).

**Table 3:** Association between the eDII and other healthy diet scores.

	Low eDII	Moderate eDII	High eDII	p-trend
Range	−9, −2	−1, +1	+2, +10	
n	439	663	362	
jMDS	6.07 ± 1.75	5.33 ± 1.71	4.95 ± 1.61	<0.001
WHO-HDI	4.21 ± 1.14	3.71 ± 1.09	3.50 ± 1.08	<0.001
AHA-HDS	3.35 ± 1.29	2.60 ± 1.21	2.23 ± 1.19	<0.001

Data are mean ± SD.

eDII, empirical Dietary Inflammatory Index; jMDS, Mediterranean diet score adapted to Japan; WHO-HDI, World Health Organization's healthy diet indicator; AHA-HDS, American Heart Association's recommended healthy diet score.

## 4 Discussion

This is the first study of a novel dietary inflammatory index reflecting for inflammatory ageing. The framework for the construction of our eDII was designed using previous dietary inflammatory indexes [3,4]. However, previous indexes were calculated using daily amounts of food or nutrient consumption, which is difficult to calculate in daily clinical practice [3,4]. In contrast, the eDII is based solely on the frequency of consumption of various foods. Therefore, most questions are easy to answer, and the index allows for a rapid estimation of dietary inflammatory potential without the need for detailed dietary records.

Our study has several limitations. First, the eDII was not construct validated using circulating inflammatory biomarkers. However, circulating biomarkers only represent the current inflammatory status. Because the eDII is expressed by daily eating habits, the validation test may be more reasonable when compared to the IAD score. In the present study, a moderate association between eDII and IAD scores was confirmed. Second, our index was empirically based to construct the scoring system; therefore, we must consider whether the trichotomous cut-offs (0, 1, and 2 points) are the best way to assess dietary factors.

## 5 Conclusions

The eDII is an easy and valid instrument to assess the inflammatory potential of dietary factors. This index is easy to use and does not require detailed estimations of nutrient intake.

## Ethical approval

The Institutional Review Board of Kio University approved the study protocol (reference number, H29-11).

## Sources of funding

This work was supported by JSPS KAKENHI Grant Number 17K09339.

## Author contribution

Masao Kanauchi: study design, data collection, data analysis, writing, final layout.

Mitsuru Shibata: data collection, data analysis.

Masaki Iwamura: data collection, data analysis.

## Conflicts of interest

None.

## Registration registration number

Name of the registry: University hospital Medical Information Network (UMIN).

Unique Identifying number or registration ID: UMIN 000027813.

Hyperlink to the registration (must be publicly accessible): https://upload.umin.ac.jp/cgi-open-bin/icdr/ctr_view_cb.cgi?recptno=R000031867&flwp_key=100438SOpdrW4hX0zt2qHxU5.

## Guarantor

Masao Kanauchi.

## Provenance and peer review

Not commissioned, externally peer reviewed.

## References

[1] Minihane A.M., Vinoy S., Russell W.R., Baka W.P., Roche H.M., Tuohy K.M., Teeling J.L., Blaak E.E., Fenech M., Vauzour D., McArdle H.J., Kremer B.H.A., Sterkman L., Vafeiadou K., Benedetti M.M., Williams C.M., Calder P.C. (2015). Low-grade inflammation, diet composition and health. Br. J. Nutr..

[2] Barbaresko J., Koch M., Schulze M.B., Nothlings U. (2013). Dietary pattern analysis and biomarkers of low-grade inflammation: a systematic literature review. Nutr. Rev..

[3] Shivappa N., Steck S.E., Hurley T.G., Hussey J.R., Hebert J.R. (2014). Designing and developing a literature-derived, population-based dietary inflammatory index. Public Health Nutr..

[4] Tabung F.K., Smith-Warner S.A., Chavarro J.E., Wu K., Fuchs C.S., Hu F.B., Chan A.T., Willett W.C., Giovannucci E. (2016). Development and validation of an empirical dietary inflammatory index. J. Nutr..

[5] Bach-Faig A., Berry E.M., Lairon D., Reguant J., Trichopoulou A., Dernini S., Medina F.X., Battino M., Belahsen R., Miranda G., Serra-Majem L. (2011). Mediterranean diet pyramid today. Public Health Nutr..

[6] D'Alessandro A., De Pergola G. (2014). Mediterranean diet pyramid: a proposal for Italian people. Nutrients.

[7] Mostofsky E., Chahal H.S., Mukamal K.J., Rimm E.B., Mittleman M.A. (2016). Alcohol and immediate risk of cardiovascular events: a systematic review and dose-response meta-analysis. Circulation.

[8] Kanauchi M., Kanauchi K. (2016). Development of a Mediterranean diet score adapted to Japan and its relation to obesity. Food Nutr. Res..

[9] Kanauchi M., Kanauchi K. (2018). The World health Organization's healthy diet indicator ad its associated factors. Prev. Med. Rep..

[10] Kanauchi M., Kanauchi K. (2018). Adherence to the American Heart Association recommended healthy diet and prevalence of metabolic syndrome in male Japanese workers. J. Nutr. Health Food Sci..

